# A prospective, randomized study of inhaled prostacyclin versus nitric oxide in patients with residual pulmonary hypertension after pulmonary endarterectomy

**DOI:** 10.1007/s11748-016-0724-2

**Published:** 2016-10-25

**Authors:** Shinichiro Abe, Keiichi Ishida, Masahisa Masuda, Hideki Ueda, Hiroki Kohno, Kaoru Matsuura, Yusaku Tamura, Michiko Watanabe, Goro Matsumiya

**Affiliations:** 1 Department of Cardiovascular Surgery, Graduate School of Medicine, Chiba University, 1-8-1 Inohana, Chuo-ku, Chiba, 260-0856 Japan; 2 Department of Cardiovascular Surgery, Chiba Medical Center, 4-1-2 Tsubakimori, Chuo-ku, Chiba, 260-0042 Japan

**Keywords:** Chronic thromboembolic pulmonary hypertension, Pulmonary endarterectomy, Residual pulmonary hypertension, Inhaled prostacyclin, Inhaled nitric oxide

## Abstract

**Objectives:**

Pulmonary endarterectomy (PEA) is an effective treatment for chronic thromboembolic pulmonary hypertension (CTEPH), but postoperative residual hypertension leads to in-hospital mortality. Inhaled epoprostenol sodium (PGI_2_) and NO are administered for pulmonary hypertension after cardiothoracic surgery. This prospective study provides the first comparative evaluation of the effects of inhaled PGI_2_ and NO on pulmonary hemodynamics, systemic hemodynamics, and gas exchange in patients developing residual pulmonary hypertension after PEA.

**Methods:**

Thirteen patients were randomized to receive either NO (*n* = 6) or PGI2 (*n* = 7) inhalation when pulmonary hypertension persisted after weaning from cardiopulmonary bypass. Hemodynamic and respiratory variables were measured before inhalation of the agent (T0); 30 min (T1), 3 h (T2), and 6 h after inhalation (T3); and the next morning (T4). The NO dose was started at 20 ppm and gradually tapered until extubation, and PGI_2_ was administered at a dose of 10 ng kg^−1^ min^−1^.

**Results:**

In both groups, mean pulmonary artery pressure (PAP) and pulmonary vascular resistance (PVR) significantly decreased over time until T4 (mean PAP: *p* < 0.0001; PVR: *p* = 0.003), while mean systemic arterial blood pressure significantly increased (*p* = 0.028). There were no significant between-group differences in patient characteristics, cardiac index, left atrial pressure, or ratio of arterial oxygen tension to fraction of inspired oxygen. There were no in-hospital deaths.

**Conclusions:**

Both inhaled PGI_2_ and NO significantly reduced PAP and PVR without adverse effects on systemic hemodynamics in patients who developed residual pulmonary hypertension after PEA. Inhaled PGI_2_ can be offered as alternative treatment option for residual pulmonary hypertension.

## Introduction

Chronic thromboembolic pulmonary hypertension (CTEPH) is characterized by pulmonary artery obstruction with organized thrombus and small vascular remodeling in the muscular pulmonary arteries, leading to pulmonary hypertension and right heart failure [1]. Although medical treatment is ineffective and palliative, pulmonary endarterectomy (PEA), which aims for complete removal of organized thrombus with the vascular intima, can provide excellent long-term outcomes with substantial improvement of pulmonary hypertension and symptoms, and is thus a potentially curative treatment of CTEPH [2–4]. However, PEA has a steep learning curve and requires meticulous postoperative management. In-hospital mortality rates after PEA still remain high (up to 7%), although experienced PEA centers can provide better results [2, 3, 5, 6]. Residual pulmonary hypertension occurs in up to 10% of patients and is significantly associated with in-hospital mortality [2, 5].

Optimal treatment of residual pulmonary hypertension after PEA requires pulmonary vasodilator agents with effective reduction of pulmonary artery pressure (PAP) and improvement of hypoxemia, without worsening systemic hypotension. Inhalation of short-acting vasodilator agents, including prostacyclin (PGI_2_) [7] and nitric oxide (NO) [8], has been proposed as a treatment option for this complication. Inhaled NO has been a preferred treatment for pulmonary hypertension and right ventricular dysfunction in patients who underwent cardiac surgery [9, 10]. However, the effect of inhaled NO for residual pulmonary hypertension after PEA is still controversial [11]. A randomized control trial of inhaled NO in patients who underwent PEA demonstrated no favorable effects on reperfusion lung edema, duration of mechanical ventilation, or perioperative mortality [12]. In contrast, inhaled iloprost, an aerosolized prostacyclin analogue, significantly reduced PAP, pulmonary vascular resistance (PVR), and increased cardiac output in patients who underwent PEA [7]. These results prompted us to compare the effectiveness of both drugs on residual pulmonary hypertension after PEA. In this prospective study, we sought to evaluate the effects of inhaled PGI_2_ and NO on pulmonary hemodynamics, systemic hemodynamics, and gas exchange in patients who developed residual pulmonary hypertension after PEA.

## Materials and methods

This prospective, randomized, observational study was approved by the institutional ethics committee on human research of Chiba University, and informed consent was obtained from each patient. Consecutive patients who underwent elective PEA at our hospital between Jan 2013 and June 2014 were enrolled. Inclusion criteria of this study were patients who developed residual pulmonary hypertension immediately after weaning from cardiopulmonary bypass. Residual pulmonary hypertension was defined as mean PAP (mPAP) above 25 mmHg despite proper treatment within 30 min after weaning from cardiopulmonary bypass. Exclusion criteria were patients who had relief of pulmonary hypertension (mPAP below 25 mmHg) after PEA (*n* = 3), and those who developed excessive pulmonary hemorrhage (*n* = 2), and severe residual pulmonary hypertension requiring extracorporeal membrane oxygenation (*n* = 1). Although it was difficult to predict which patients would develop residual pulmonary hypertension, preoperative mPAP are shown to be associated with postoperative high mPAP [4]. So the objective of dynamic stratified randomization by computer is to ensure balance of the both groups with respect to preoperative mPAP and PVR. Patients were registered by the Patient Registration Center System.

### Surgery and postoperative management

All patients underwent right heart catheterization and pulmonary angiography for a definitive diagnosis of CTEPH, and operability was assessed by a CTEPH team. An inferior vena cava filter (Günther-Tulip^®^Vena Cava Filter, Cook Medical Inc., Bloomington, IN, USA) was placed prior to the surgery in all patients. PEA was performed via a median sternotomy and under periods of deep hypothermic circulatory arrest, according to a protocol of the University of California, San Diego [2]. An arterial line was placed in both the femoral artery and the radial artery to monitor systemic arterial pressure. A Swan-Ganz catheter (Baxter Healthcare Corp., Deerfield, IL) was introduced to continuously monitor pulmonary artery and central venous pressure, as well as cardiac output (Vigilance Monitor, Baxter Healthcare Corp.). During cardiopulmonary bypass, a left atrial catheter was placed via the right upper pulmonary vein to monitor left atrial pressure and administer norepinephrine to maintain systemic arterial pressure without vasoconstriction of the pulmonary arteries. Epinephrine was administered via a central venous catheter, if necessary. Catecholamine was adjusted to maintain the mean femoral arterial pressure above 60 mmHg. No vasodilator agents were intravenously administered. The baseline ventilator setting at weaning from cardiopulmonary bypass was as follows: oxygen fraction = 1.0, positive end-expiratory pressure (PEEP) = 10 cmH_2_O, and tidal volume = 6–8 mL/kg. Mechanical ventilator settings were adjusted to maintain PaO_2_ above 80 mmHg and PaCO_2_ < 40 mmHg. Patients were sedated with propofol and mechanically ventilated with high PEEP until the next morning. Negative water balance was maintained through the aggressive administration of diuretics. Reperfusion lung edema was defined as hypoxemia (PaO_2_/FiO_2_ ratio < 300) accompanied by a new lung infiltrate on chest X-ray in an area that had been reperfused, without another cause for the hypoxemia or acute chest radiograph abnormality.

### Protocols

After weaning from cardiopulmonary bypass, the preload and dose of catecholamine were optimized and ventilator stings were adjusted to treat hypoxemia and hypercapnia. If pulmonary hypertension persisted above 25 mmHg, pulmonary vasodilator inhalation therapy was indicated. Thirteen patients were randomized to receive either NO (*n* = 6) or PGI_2_ (*n* = 7) following a randomization list edited by the institutional Clinical Study Coordination Center, whose members were unaware of the patients’ identities. The patients continuously inhaled each agent until extubation or the point at which mPAP decreased below 25 mmHg. The patients were not blinded to the inhaled agent because only one drug delivery system was mounted at a time on the ventilator. The hemodynamic and respiratory variables were measured before inhalation of the agent (T0); 30 min (T1), 3 h (T2), and 6 h after inhalation (T3); and on the next morning (T4). Side effects attributable to the use of the agents were recorded, including methemoglobinemia and excessive chest drain bleeding due to platelet dysfunction. Methemoglobin was measured every 6 h with a blood oximeter (ABL 700 series; Radiometer, Copenhagen, Denmark). To avoid rebound pulmonary hypertension after the withdrawal of No inhalation, No doses were gradually decreased before extubation.

### Studied agents

PGI_2_ group: epoprostenol sodium (FLOLAN, epoprostenol sodium injection, prostacyclin; GlaxoSmithKline LLC, Brentford, United Kingdom) was continuously inhaled in a dose of 10 ng kg^−1^ min^−1^. For aerosolization of epoprostenol, we connected a jet nebulizer (AeroNeb Pro; Aerogen, Galway, Ireland) to a ventilator (Puritan BennettTM 840 Ventilator; Covidien, Minneapolis, MN). The nebulizer chamber (Cirrus; Intersurgical, Twickenham, United Kingdom) was connected to the inspiratory limb 10 cm from the endotracheal tube. We dissolved 1.5 mg of epoprostenol in glycine buffer diluent (sterile diluent for FLOLAN) to obtain a concentration of 15,000 ng/mL. Epoprostenol solution was continuously delivered at a constant rate of 20 mL/h during intubation.

NO group: NO was supplied in cylinders as 10,000 parts per million (ppm) of NO in nitrogen (Woikoski, Voikoski, Finland). We used a Nomius C blender system (Nomius C, version 1.2; Gävle, Sweden) to deliver NO into the breathing circuit of the ventilator. Inspired NO and nitrogen dioxide (NO_2_) concentration were monitored continuously using a Nomius C chemiluminescence analyzer. NO inhalation started at 20 ppm.

### Statistical analysis

The results are expressed as means ± standard deviations. Continuous variables were compared via the Mann–Whitney *U* test, and categorical data were compared via the Chi-square test. Analysis of variance for repeated measures was used to detect differences between the PGI_2_ and NO groups for the effects of drug, time, hemodynamic conditions, and respiratory conditions. In case of a significant interaction, time × group comparison was performed using Tukey’s method. Differences were considered significant for two-sided* p* values less than 0.05. All statistical analyses were performed using JMP^®^ 12 (SAS Institute Inc., Cary, NC).

## Results

Baseline patient characteristics are shown in Table 1. There were no significant differences in preoperative pulmonary hemodynamics or hypoxemia between the two groups. Mean duration of cardiopulmonary bypass (PGI_2_: 352 ± 67 versus NO: 347 ± 20 min; *p* = 0.87) and deep hypothermic circulatory arrest (PGI_2_: 47 ± 9 versus NO: 47 ± 11 min; *p* = 0.92) were also comparable. There was no in-hospital mortality and no patient developed methemoglobinemia, excessive chest drain bleeding, rebound pulmonary hypertension, or systemic hypotension requiring cessation of the drugs during the study period. However, 2 patients (1 patient in each group) developed late tamponade. Extubation was performed on postoperative day 2 for 2 patients in the PGI_2_ group who had excessive positive balance during the postoperative period, as well as one patient who developed reperfusion lung edema in the NO group. The other patients were extubated on postoperative day 1.

**Table 1:** Preoperative patient characteristics

	PGI_2_ (*n* = 6)	NO (*n* = 7)	*p* value
Female (number)	6	7	
Age (years)	70 ± 2	64 ± 7	0.101
BSA (m^2^)	1.3 ± 0.1	1.4 ± 0.1	0.259
NYHA functional class (no. II/III/IV)	2/3/1	3/3/1	
CI (L min^−1^ m^−2^)	2.4 ± 1.1	2.7 ± 0.5	0.653
mPAP (mmHg)	45 ± 6	51 ± 5	0.466
mBP (mmHg)	71 ± 5	76 ± 12	0.442
PVR (dyne s cm^−5^)	756 ± 174	725 ± 236	0.810
SVR (dyne s cm^−5^)	1931 ± 566	1702 ± 432	0.462
PaO_2_ (mmHg)	52 ± 8	53 ± 5	0.785
PaO_2_/FIO_2_ ratio	279 ± 52	239 ± 86	0.785

Values are shown as mean ± standard deviations

*BSA* body surface area,* NYHA* New York Association,* CI* cardiac index,* mPAP* mean pulmonary arterial pressure,* mBP* mean blood pressure,* PVR* pulmonary vascular resistance,* SVR* systemic vascular resistance,* 6MWD* 6 minutes walk distance,* PaO*_2_ /*FIO*_2_ *ratio* ratio of arterial oxygen tension to fraction of inspired oxygen

**Fig. 1:**
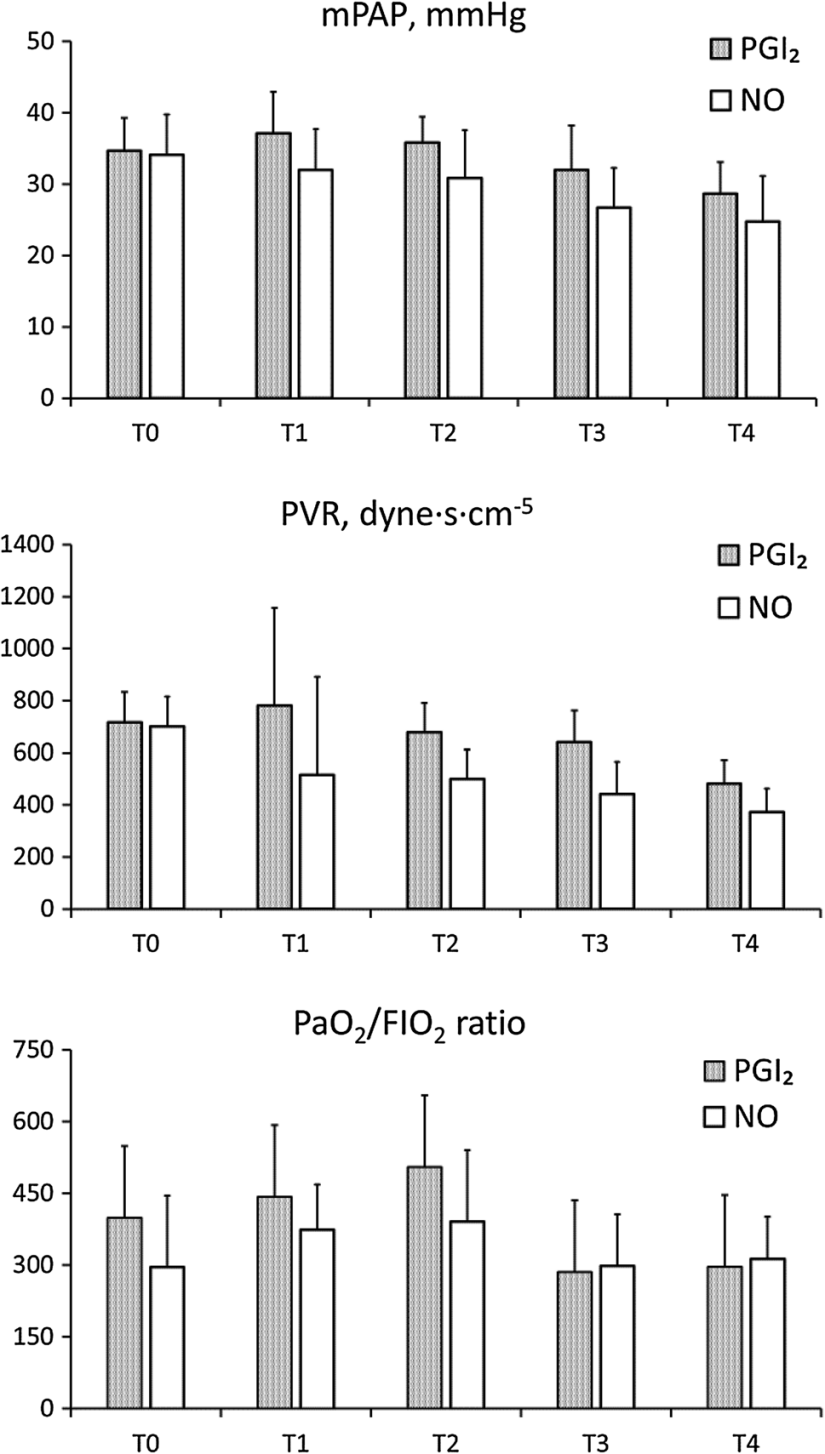
Changes in mean pulmonary artery pressure (mPAP), pulmonary vascular resistance (PVR) and ratio of arterial oxygen tension to fraction of inspired oxygen (PaO_2_/FIO_2_ ratio) at each time point in the PGI_2_ (*mesh*) and No groups (*white*). Variables were measured before inhalation of the agent (T0); 30 min (T1), 3 h (T2), and 6 h after inhalation (T3); and the next morning (T4)

**Fig. 2:**
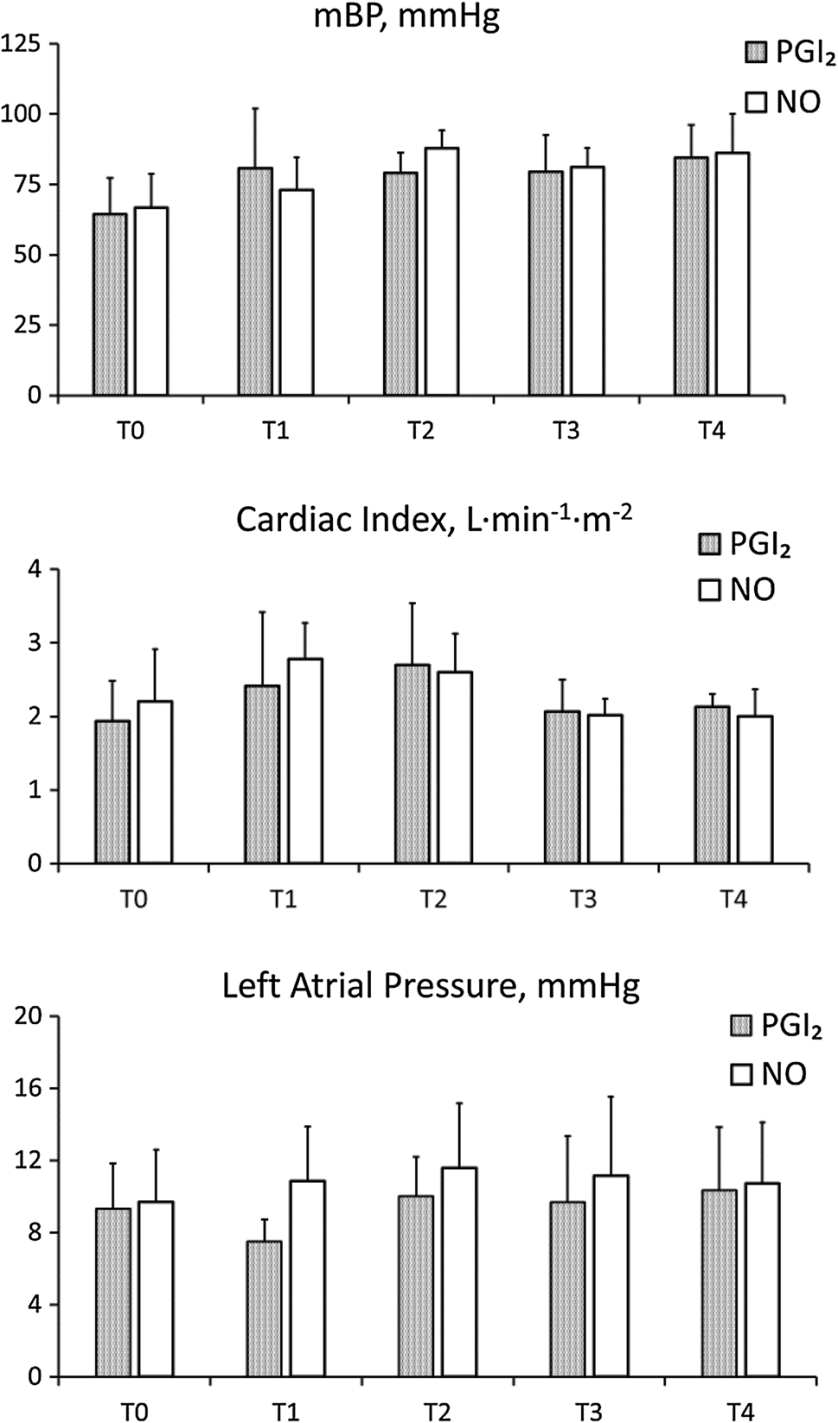
Changes in systemic hemodynamics: mean systemic arterial blood pressure (mBP), cardiac index and left atrial pressure at each time point in the PGI_2_ (*mesh*) and NO groups (*white*). Variables were measured before inhalation of the agent (T0); 30 min (T1), 3 h (T2), and 6 h after inhalation (T3); and the next morning (T4)

The figures show the pulmonary hemodynamics, PaO_2_/FiO_2_ ratio (Fig. 1) and systemic hemodynamics (Fig. 2) during the study period. Mean PAP (PGI2: 34.7 ± 5.0 versus NO: 34.1 ± 6.0 mmHg; *p* = 0.87) and PVR (PGI2: 717 ± 127 versus NO: 699 ± 354 dyne s cm^−5^; *p* = 0.91) at T0 were comparable between the groups. After induction of the agents, mPAP and PVR significantly decreased over time until T4 (mPAP: *p* < 0.0001 and PVR: *p* = 0.003), although there were no significant statistical differences between the groups. Mean PAP and PVR at T4 were similar in both groups (PGI_2_: 28.7 ± 4.8 versus NO: 24.7 ± 6.9 mmHg; *p* = 0.27, and PG: 481 ± 98 versus NO: 373 ± 147 dyne s cm^−5^; *p* = 0.15). Despite residual pulmonary hypertension at the time of weaning from cardiopulmonary bypass, 2 patients in the PGI2 group and 4 patients in the NO group had no residual pulmonary hypertension the next morning. The PaO2/FiO2 ratio in the PG group appeared to be higher at T2, but the difference was not statistically significant (Fig. 1). Mean systemic pressure (mBP) significantly increased over time until T4 (*p* = 0.028). Cardiac index and left atrial pressure were stable and comparable between the groups during the study period (Fig. 2). Water balance was compared during the surgery and the postoperative period, which lasted from admission to the intensive care unit until the next morning. Patients in the No group had a trend toward having a more positive water balance during the surgery (PGI_2_: 3074 ± 1523 versus No: 5633 ± 2923 mL; *p* = 0.08). However, there was no statistically significant difference between the groups on the morning of the next day (PGI_2_: 185 ± 1045 versus No: −471 ± 1028 mL; *p* = 0.27).

There were no statistically significant differences between the groups at 1 year after the surgery: mPAP (PGI_2_: 28.0 ± 3.6 versus NO: 28.4 ± 3.3 mmHg; *p* = 0.93), cardiac index (PGI_2_: 2.96 ± 0.1 versus NO: 2.7 ± 0.1 mmHg; *p* = 0.26) and PVR (PGI_2_: 399.3 ± 68.1 versus NO: 360.4 ± 63.1 dyne s cm^−5^; *p* = 0.68). All patients in both groups preoperatively administered oral PH drug: PDE5 inhibitors, ET receptor antagonists and prostanoids. Four patients (PGI_2_: 3 patients versus NO: 1 patient) postoperatively improved mPAP and discontinued medications; however, the others were postoperatively treated with soluble guanylate cyclase. There were no patients treated with BPA in both groups.

## Discussion

The leading cause of in-hospital mortality after PEA is residual pulmonary hypertension, which can be attributed to several pathological factors, including residual thrombus due to incomplete PEA, small vessel disease in the muscular arteries, induction of inflammatory reaction, and ischemia reperfusion injury to the lung [13, 14]. Patients may develop residual pulmonary hypertension even in cases of successful PEA, which aims at complete removal of all organized thrombus in the segmental and subsegmental arteries. Among the factors that may be responsible for residual pulmonary hypertension, small vessel disease has an important relationship with the structural pathology of CTEPH. Small vessel disease is caused by chronic exposure of vascular components to high pressure and shear stress, and can be responsible for residual pulmonary hypertension after PEA [15]. PEA is an invasive procedure that requires a prolonged duration of cardiopulmonary bypass and periods of deep hypothermic circulatory arrest. Surgical trauma and cardiopulmonary bypass induce inflammatory reactions by activating inflammatory cytokines and vasoconstrictive mediators. In addition, removal of the organized thrombus causes ischemia reperfusion injury to the lung, and the inflammatory reaction further exacerbates the injury and progresses to reperfusion lung edema [13]. In particular, in cases with less than mild to moderate residual pulmonary hypertension, pulmonary vasodilator medicines can exacerbate lung edema; however, inhalation therapy with NO or PGI_2_ did not adversely affect reperfusion lung edema, since this study showed improved P/F ratio at T1 and T2 in both groups. Furthermore, to prevent reperfusion lung edema, we avoided high cardiac output by reducing cardiac preload, which sometimes have resulted in systemic hypotension. We attempted at maintaining mBP above 60 mmHg by means of noradrenaline administration. Patients in PGI_2_ had lower LAP and higher mBP at T1 compared with the baseline, which may indicate low cardiac preload and vasoconstriction due to noradrenaline.

Inhaled NO has been the preferred treatment in patients with pulmonary hypertension who undergo cardiothoracic surgery [9, 10, 16, 17]. However, there is some controversy regarding whether inhaled NO is clinically effective after PEA. A randomized control trial of inhaled NO did not reduce reperfusion lung edema, the duration of mechanical ventilation, or perioperative mortality [12]. In contrast, another crossover study of inhaled NO in patients who underwent PEA showed significant improvements of mPAP (baseline: 22.8 ± 6.2 versus during NO: 21.2 ± 5.0 mmHg; *p* < 0.05) and PVR index (baseline: 312 ± 98 versus during NO: 277 ± 93 dyne s cm^−5^; *p* < 0.01) 12 h after the surgery, although there were no significant differences 3 h after the surgery [8]. On the other hand, inhaled PGI_2_ has been examined extensively in light of the disadvantages of inhaled NO, and favorable evidence has been obtained. Wet et al. showed a significant decrease in mPAP from 35 ± 9 to 24 ± 8.4 mmHg 6 h after the induction of continuous inhalation of epoprostenol in patients who underwent cardiothoracic surgery [18]. Hache et al. showed that single inhalation of epoprostenol significantly decreased mPAP from 32 ± 9 to 28 ± 8 mmHg in patients with pulmonary hypertension undergoing cardiac surgery [19]. In patients who underwent PEA, Kramm et al. showed that a high dose of inhaled iloprost significantly decreased mPAP as compared with placebo, with a mean decrease of 11 ± 1.1 mmHg [7].

The present study showed that both inhaled PGI2 and NO reduced PAP and PVR without leading to deteriorated systemic circulation, thus indicating that inhaled PGI2 can be an alternative agent to NO in the treatment of residual pulmonary hypertension after PEA. Previous studies evaluating the effects of inhaled PGI2 and NO demonstrated that the outcomes of inhaled PGI_2_ were similar to or even better than the outcomes of NO [20–22]. A randomized crossover study revealed favorable effects of both inhaled epoprostenol and NO on pulmonary hemodynamics and cardiac output in heart and lung transplant patients [21]. In another randomized study of inhaled iloprost and NO in patients with pulmonary hypertension who underwent cardiac surgery, both agents significantly reduced mPAP, reduced PVR, and increased cardiac output, but inhaled iloprost showed significant greater improvements [22]. In addition, the combination therapy of inhaled NO and PGI_2_ has additive effects on pulmonary hemodynamics [23]. A successful combination therapy was also reported in a case of residual pulmonary hypertension after PEA [24].

The main limitation of this study is the small number of analyzed patients, which is attributable to the relative rarity of the operation. Furthermore, this study is not a confirmatory phase III trial, but an exploratory randomized phase II trial. The purpose of the trial is to evaluate the safety and efficacy and to determine whether to perform a subsequent phase III trial. Although the number of our patients enrolled in this study was small, we conducted a post hoc power analysis; with *n* = 16 patients, the study was designed to detect differences in mean values corresponding to an effect size of 1.1 with a power of 80% when applying the Student’s *t* test at a significance level of 0.20. In addition, the present study did not include a placebo group or a dose–response assessment for different doses of PGI2 and NO; these additional investigations were not undertaken because residual pulmonary hypertension is associated with high in-hospital mortality after PEA. The dose of epoprostenol (10 ng kg^−1^ min^−1^) in this study might have been smaller than the doses in previous studies (160,000 ng/h), while the dose of inhaled NO (20 ppm) was consistent with previous studies [18, 21]. However, no dose-dependent effect of inhaled epoprostenol (0–50 ng/kg/min) on pulmonary hemodynamics was demonstrated in a study of patients with acute respiratory distress syndrome, while the PaO_2_/FiO_2_ ratio improved with the increased dose [25]. Finally, the precise amount of epoprostenol that reaches the alveoli is uncertain because of losses in the nebulizer chamber and ventilator tubing, and may vary between patients.

## Conclusion

This prospective, randomized study of inhaled pulmonary vasodilators demonstrated that both inhaled PGI2 and NO significantly reduced PAP and PVR without adverse effects on systemic hemodynamics in patients who developed residual pulmonary hypertension after PEA. Therefore, inhaled PGI2 can be offered as alternative treatment option for residual pulmonary hypertension.
